# Lymphoma tumor burden before chimeric antigen receptor T-Cell treatment: RECIL vs. Lugano vs. metabolic tumor assessment

**DOI:** 10.3389/fonc.2022.974029

**Published:** 2022-09-08

**Authors:** Michael Winkelmann, Veit L. Bücklein, Viktoria Blumenberg, Kai Rejeski, Michael Ruzicka, Marcus Unterrainer, Christian Schmidt, Franziska J. Dekorsy, Peter Bartenstein, Jens Ricke, Michael von Bergwelt-Baildon, Marion Subklewe, Wolfgang G. Kunz

**Affiliations:** ^1^ Department of Radiology, University Hospital, Ludwig Maximilian University (LMU) Munich, Munich, Germany; ^2^ Department of Medicine III, University Hospital, Ludwig Maximilian University (LMU) Munich, Munich, Germany; ^3^ Department of Nuclear Medicine, University Hospital, Ludwig Maximilian University (LMU) Munich, Munich, Germany; ^4^ ^LMU^ Comprehensive Cancer Center München-LMU (CCCM), Ludwig Maximilian University (LMU) Munich, Munich, Germany

**Keywords:** CAR T-cell therapy, Lugano criteria, RECIL, MTV, tumor burden assessment, PET/CT (18)F-FDG

## Abstract

**Purpose:**

High tumor burden has emerged as a negative predictor of efficacy in chimeric antigen receptor T-cell therapy (CART) in patients with refractory or relapsed large B-cell lymphoma. This study analyzed the deviation among imaging-based tumor burden (TB) metrics and their association with progression-free (PFS) and overall survival (OS).

**Materials and methods:**

In this single-center observational study, we included all consecutively treated patients receiving CD19 CART with available baseline PET-CT imaging. Imaging-based TB was determined based on response evaluation criteria in lymphoma (RECIL), the Lugano criteria, and metabolic tumor volume. Total, nodal and extranodal TB were represented, according to the respective criteria, by sum of longest diameters (TB_RECIL_), sum of product of perpendicular diameters (TB_Lugano_), and metabolic tumor volume (TB_MTV_). Correlation statistics were used for comparison. Proportional Cox regression analysis studied the association of TB metrics with PFS and OS.

**Results:**

34 consecutive patients were included (median age: 67 years, 41% female) with total median baseline TB_RECIL_ of 12.5 cm, TB_Lugano_ of 4,030 mm^2^ and TB_MTV_ of 330 mL. The correlation of TB_RECIL_ and TB_Lugano_ with TB_MTV_ was strong (ρ=0.744, p<0.001 and ρ=0.741, p<0.001), with lowest correlation for extranodal TB_RECIL_ with TB_MTV_ (ρ=0.660, p<0.001). Stratification of PFS was strongest by total TB_MTV>50%_ (HR=2.915, p=0.042), whereas total TB_RECIL>50%_ and total TB_Lugano>50%_ were not significant (both p>0.05). None of the total TB metrics were associated with OS (all p>0.05).

**Conclusion:**

Pre-CART TB metrics vary significantly based on the assessment method, impacting their association with survival outcomes. The correlation between TB_RECIL_, TB_Lugano_ and TB_MTV_ was influenced by disease phenotype and prior bridging therapy. TB method of assessment must be considered when interpreting the impact of TB on outcomes in clinical trials. Considering the heterogeneity, our results argue for standardization and harmonization across centers.

## Introduction

Chimeric antigen receptor T-cell therapy (CART) targeting the CD19 antigen (1) prolongs progression-free survival (PFS) and overall survival (OS) in relapsed or refractory diffuse large B-cell lymphoma (DLBCL; 2–4), follicular lymphoma (FL; 3, 4), and mantle cell lymphoma (MCL; 5). Tumor imaging is primarily used for response assessment, yet mounting evidence suggests that tumor burden (TB) not only represents a prognostic biomarker at baseline (5–8), but also a means to dynamically assess disease response in the context of CD19 CART (9, 10).

Imaging is routinely performed using positron emission tomography-computed tomography (PET-CT) with the tracer 18F-fluorodeoxyglucose (18F-FDG) or computed tomography (CT). In current and ongoing phase III trials, the most widely adopted assessment is based on the Lugano criteria from 2014 (11, 12). In recent years, the simplified response evaluation criteria in lymphoma (RECIL; 13) have been proposed. RECIL relies on unidimensional measurement of ≤3 manifestations, whereas Lugano criteria apply bidimensional measurements of ≤6 lesions. This routine clinical trial documentation can be used as surrogate of the lymphoma TB.

Quantifying the entire lymphoma TB requires methods that are not routinely performed in clinical practice or within clinical trials. PET imaging facilitates TB assessment based on the high tumor-to-background ratio of hypermetabolic lymphomas. This enables measurement of the (entire) metabolic tumor volume (MTV; only limited by PET spatial resolution which is negligible for most lymphoma phenotypes). This approach, however, has limitations in anatomical locations with high physiologic metabolic activity or tracer excretion, such as the central nervous system or the kidneys.

The influence and heterogeneity of TB assessment methods on post-CART outcomes remains poorly understood. The most important and distinct differences among these methods of assessment are provided in **Table 1**. Examples of subgroup analysis from recent clinical trials and the applied TB methods are illustrated in **Table 2**. Understanding the differences between TB assessment methods would enable standardization/harmonization in the context of clinical trials and high-quality real-world evidence. This may support the development of TB metrics for improved outcome prediction.

**Table 1:** Methods of Tumor Burden Assessments.

TB Method	Unit	Number of TL	Technique		Software
RECIL	cm	≤3	Sum of longest diameter (SLD) of up to 3 target lesions (nodal and/or extranodal)	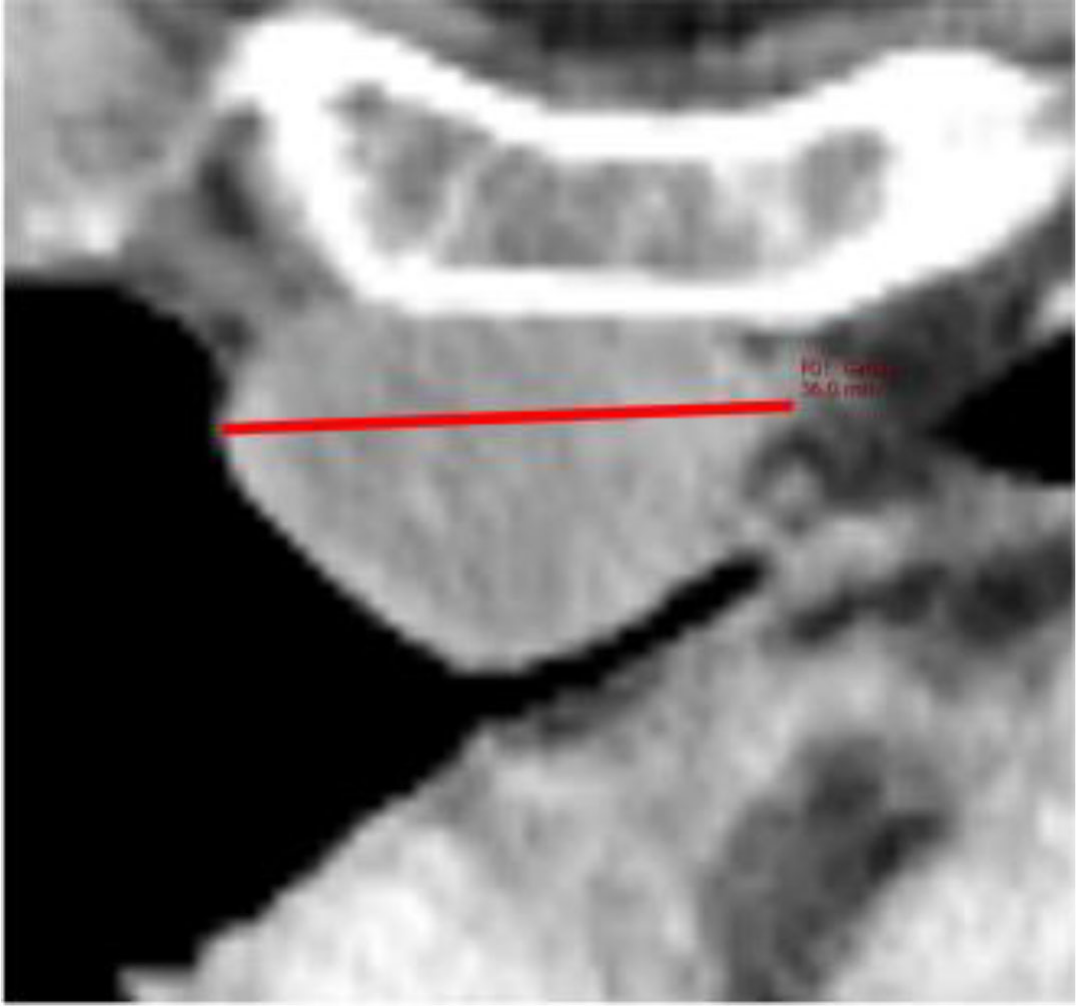	Mint Lesion
Lugano	mm^2^	≤6	Sum of perpendicular diameter (SPD) of up to 6 target lesions (nodal and/or extranodal)	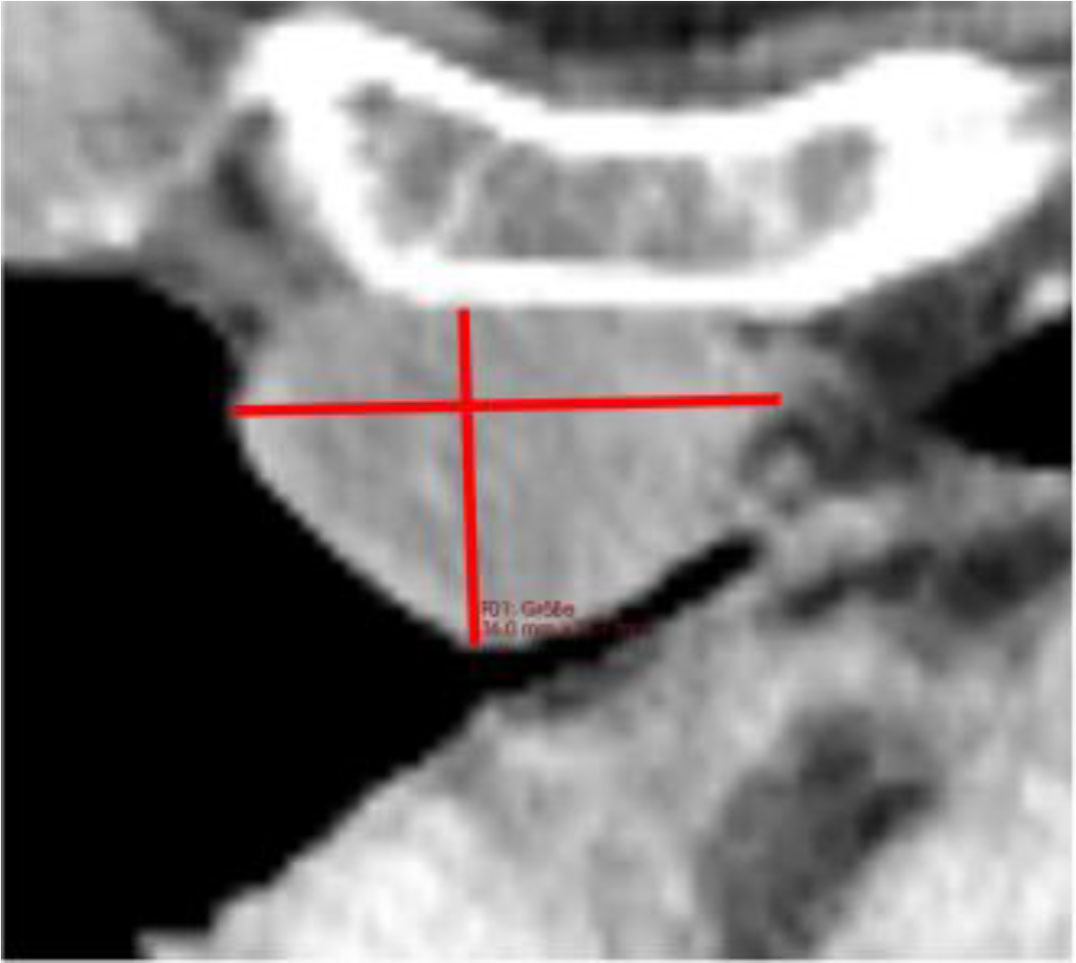	Mint Lesion
MTV	mL	unlimited	Semi-automatic quantification of all hypermetabolic TB with SUV≥4	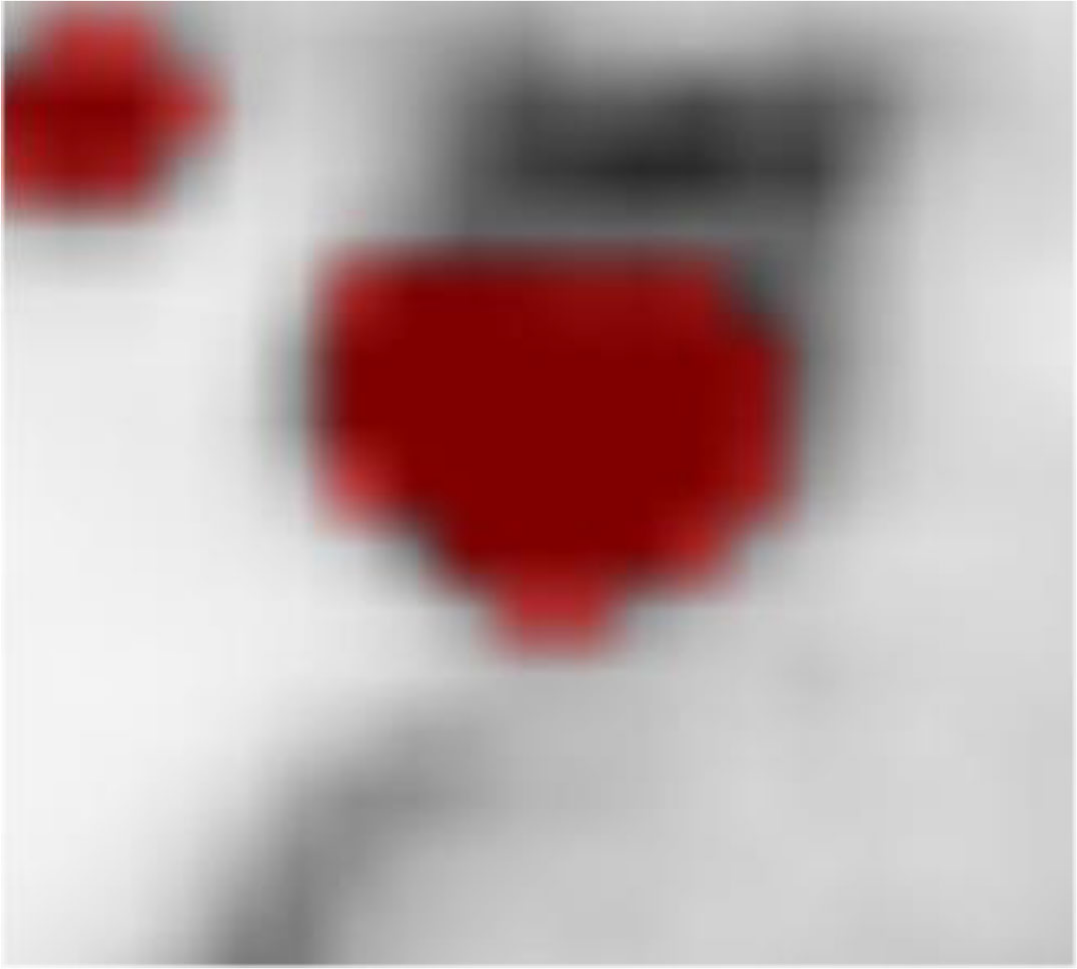	LIFEx

MTV, metabolic tumor volume; RECIL, response evaluation criteria in lymphoma; SUV, standardized uptake value; TB, tumor burden; TL, target lesions.

**Table 2:** Reported Tumor Burden Metrics in Recently Published CART Trials.

Subtype	TB Method	Reported TB Metric	Impact or Application	Trial and Reference
DLBCL	MTV	100 mL (cutoff)	ΔORR=16%	JULIET/Schuster et al. NEJM (2)
DLBCL	Cheson*	SPD (continuous)	Low TB strong predictor of durable response	ZUMA-1/Locke et al. Blood Adv (6)
MCL	Lugano	SPD (median)	ΔORR=11% ΔOngoing RR=24%	ZUMA-2/Wang et al. NEJM (14)
DLBCL, HGBL	Lugano	SPD (median)	Study arms balanced regarding TB	ZUMA-7/Locke et al. NEJM (15)
DLBCL	Lugano	SPD (quartiles)	Higher peak expansion of CART cells in Q2 & Q3	ZUMA-12/Neelapu et al. Nat Med (16)
HL	MTV	60 mL (cutoff)	ΔPFS=13.3 months ΔPFS@1Y=44%	NCT02690545/Voorhees et al. Blood Adv (17)

* The Cheson TB method can be considered identical to the Lugano TB method. DLBCL, diffuse large B-cell lymphoma; HGBL, high-grade B-cell lymphoma; HL, Hodgkin’s lymphoma; MCL, mantle cell lymphoma; MTV, metabolic tumor volume; N/A, not available; ORR, overall response rate; PFS, progression-free survival; SPD, sum of product of diameters; TB, tumor burden.

We therefore studied the deviation among imaging-based TB metrics, reasons for non-conformity, and the association of different TB metrics with PFS and OS.

## Material and methods

### Study design and population

The study population was based on a prospective registry of all consecutive patients who were treated at the Comprehensive Cancer Center Munich-Ludwig-Maximilian University Munich (CCCM^LMU^) with commercial CART products in between Jan. 2019 and Feb. 2022. The following inclusion criteria were applied:

1. Patients with refractory or relapsed lymphoma (DLBCL and MCL)
2. Available PET-CT imaging studies at baseline (≤2 weeks before CART)
3. Any measurable disease by either morphologic and/or metabolic imaging according to Lugano criteria (11)

The following exclusion criteria were applied:

1. Any non-diagnostic imaging studies

Patients received lymphodepletion with fludarabine and cyclophosphamide according to the manufacturers’ instructions. Bridging therapy was defined as systemic therapy between time of indication and CART transfusion. Serum levels of lactate dehydrogenase (LDH) were determined at the time of apheresis and before lymphodepletion. Immunotoxicity was graded according to American Society for Transplantation and Cellular Therapy (ASTCT) consensus criteria.

### 18F-FDG PET/CT imaging

PET/CT images were acquired approximately 45 min after tracer injection (159-275 MBq weight-adapted with approximately 2.5–4.5 MBq 18F-FDG per kg bodyweight) and for the FDG PET/CT contrast-enhanced or unenhanced CTs using a slice thickness of 2 mm 120 kVp, 100–400 mAs, and dose modulations were performed for attenuation correction. The following scanners were used: Biograph 64 and Biograph mCT (Siemens Healthineers, Germany) or Discovery 690 (GE Healthcare, USA). Both scanners fulfilled the requirements indicated in the European Association of Nuclear Medicine (EANM) imaging guidelines and obtained EANM Research Ltd. (EARL1) accreditation during acquisition. The following reconstruction algorithms were used: Biograph 64: TrueX (3 iterations, 21 subsets) with Gaussian post-reconstruction smoothing (2 mm full width at half-maximum). Biograph mCT: TrueX (3 iterations, 21 subsets). Discovery 690: VUE Point Fx algorithm with 2 iterations and 36 subsets. All systems resulted in a PET image with a voxel size of 2 × 2 × 2 mm^3^. Images were normalized to decay corrected injected activity per kg body weight (SUV g/ml).

### Imaging assessment

For RECIL, ≤3 lesions were assessed using the sum of longest diameters (SLD) as TB metric. If available, the dominant extranodal lesions were included. For Lugano, ≤6 lesions were assessed using the sum of the product of diameters (SPD). If available, the dominant extranodal lesions were included. All imaging analyses for structured RECIL and Lugano assessment were performed with dedicated trial reporting software mintLesion 3.8 (mint Medical GmbH; Heidelberg, Germany). The MTV was determined as follows: Attenuation corrected PET images were analyzed by using the open-source software platform LIFEx (https://www.lifexsoft.org; 18) with an absolute threshold of standardized uptake value (SUV) ≥4 to define hypermetabolic lymphoma tissue as described before (19, 20); these studies have also demonstrated reproducibility of this approach. A focal metabolic increase with SUVmax ≥4 and a morphologic correlate on the CT scan and/or biopsy confirmation was considered to define bone involvement. A reader with 5 years of experience in radiology and nuclear medicine performed the initial manual correction, which was subsequently reviewed by a senior physician with 8 years of experience in radiology and nuclear medicine.

### Survival analysis

PFS was defined as the time from initiation of CART treatment to progression of lymphoma based on imaging, unequivocal clinical findings, and/or histologic confirmation. OS was defined as the time from the start of therapy to death from any cause. For survival analysis, PFS and OS were visualized using Kaplan-Meier survival curves with dichotomization for median TB_RECIL_, TB_Lugano_ and TB_MTV_. Log-rank (Mantel-Cox) test was performed to compare survival curves and calculate hazard ratios.

### Statistical analysis

All statistical analysis were performed using GraphPad Prism 7. Both D’Agostino-Pearson and Kolmogorov-Smirnov test were used to assess normal distribution of each TB quantification method. Due to the lack of normal distribution of TB_MTV_ and TB_Lugano_, nonparametric Spearman correlation was performed to evaluate the relationship between the different methods of TB assessment. The 95% confidence interval (CI) is shown in brackets after Spearman’s ρ. To test the significance of the results, the p-value associated with the correlation coefficient was analyzed and a p<0.05 was considered significant. The relationship between X and Y was summarized by a fitted regression line on the graphs.

## Results

### Patient characteristics

**Figure 1:**
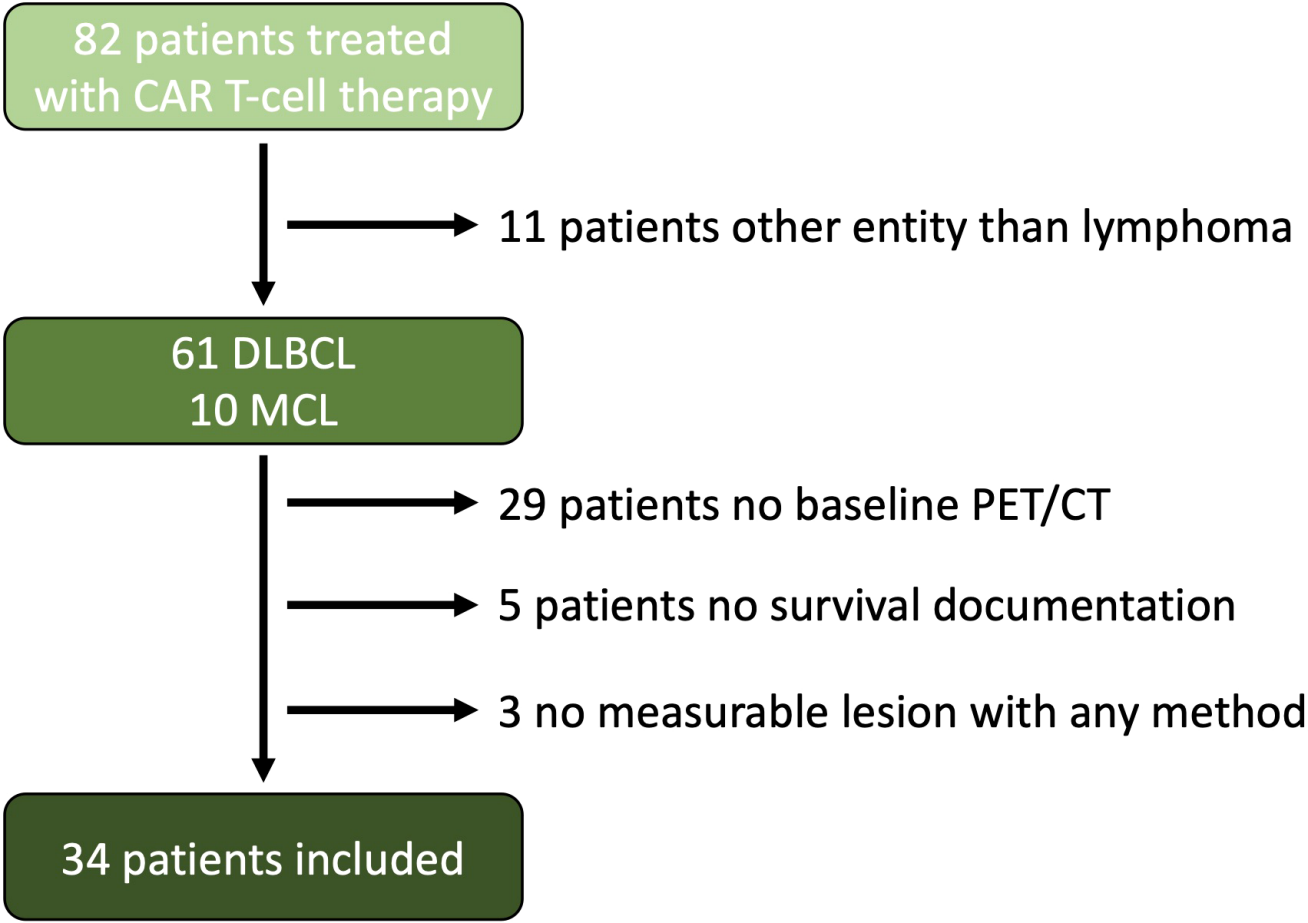
Flow Chart. A total of 82 patients were treated with CAR T-cell therapy at our site starting from Jan. 2019 to Feb. 2022. 11 patients were excluded because of entities other than lymphoma, 29 patients did not have a baseline PET/CT examination near before CAR T-cell transfusion, and 5 patients were excluded because of lack of survival documentation, and 3 patients did not have a measurable lesion according to the Lugano criteria or a hypermetabolic lesion.

Thirty-four patients matched the inclusion criteria (median age: 67 years, 41% female). A flow chart is provided in **Figure 1**. 26 patients presented with DLBCL and 8 patients with MCL. 20 patients received tisagenlecleucel, 8 brexucabtagene autoleucel/KTE-X19, 5 axicabtagene ciloleucel, and 1 Lisocabtagene maraleucel. The median total baseline TB_RECIL_ was 12.5 cm, TB_Lugano_ 4,030 mm^2^ and TB_MTV_ 330 mL. The median nodal and extranodal baseline TB_RECIL_ was 8.5 and 0.8 cm, TB_Lugano_ 2 125 mm^2^ and 282 mm^2^, TB_MTV_ 51 mL and 24 mL. The patient characteristics are shown in **Table 3**.

**Table 3:** Patient Characteristics.

Age	median	67
**Gender**	Female: Male:	14 (41%) 20 (59%)
**Lymphoma**	DLBCL: MCL:	26 (76%) 8 (24%)
**Ann Arbor Stage**	I: II: III: IV:	2 (5,9%) 4 (11,8%) 7 (20,6%) 21 (61,8%)
**IPI**	1: 2: 3: 4: 5:	6 (17,6%) 8 (23,5%) 8 (23,5%) 8 (23,5%) 4 (11,8%)
**CAR T Product**	Tisagenlecleucel: Brexucabtagene autoleucel: Axicabtagene ciloleucel: Lisocabtagene maraleucel:	20 (58,8%) 8 (23,5%) 5 (14,7%) 1 (2,9%)
**Bridging**	(R)-Pola-Benda (R)-DHAP (R)-GemOx Radiation (R)-Pola (R)-Pixantrone (R)-Dexa-Cyclo Ibrutinib Nivolumab Venetoclax No bridging	8 6 5 2 1 1 1 1 1 1 7
**TB_RECIL_ (median)**	Total: Nodal: Extranodal:	12.5 cm 8.5 cm 0.8 cm
**TB_Lugano_ (median)**	Total: Nodal: Extranodal:	4,030 mm^2^ 2,125 mm^2^ 282 mm^2^
**TB_MTV_ (median)**	Total: Nodal: Extranodal:	330 mL 51 mL 24 mL
**LDH (median)**	Apheresis Prior Lymphodepletion	356 U/L 296 U/L

CAR; chimeric antigen receptor; DLBCL, diffuse large B cell lymphoma; IPI, International Prognostic Index; LDH, lactate dehydrogenase; MCL, mantle cell lymphoma; MTV, metabolic tumor volume; RECIL, response evaluation criteria in lymphoma; TB, tumor burden.

### Correlation analysis among TB metrics

**Figure 2:**
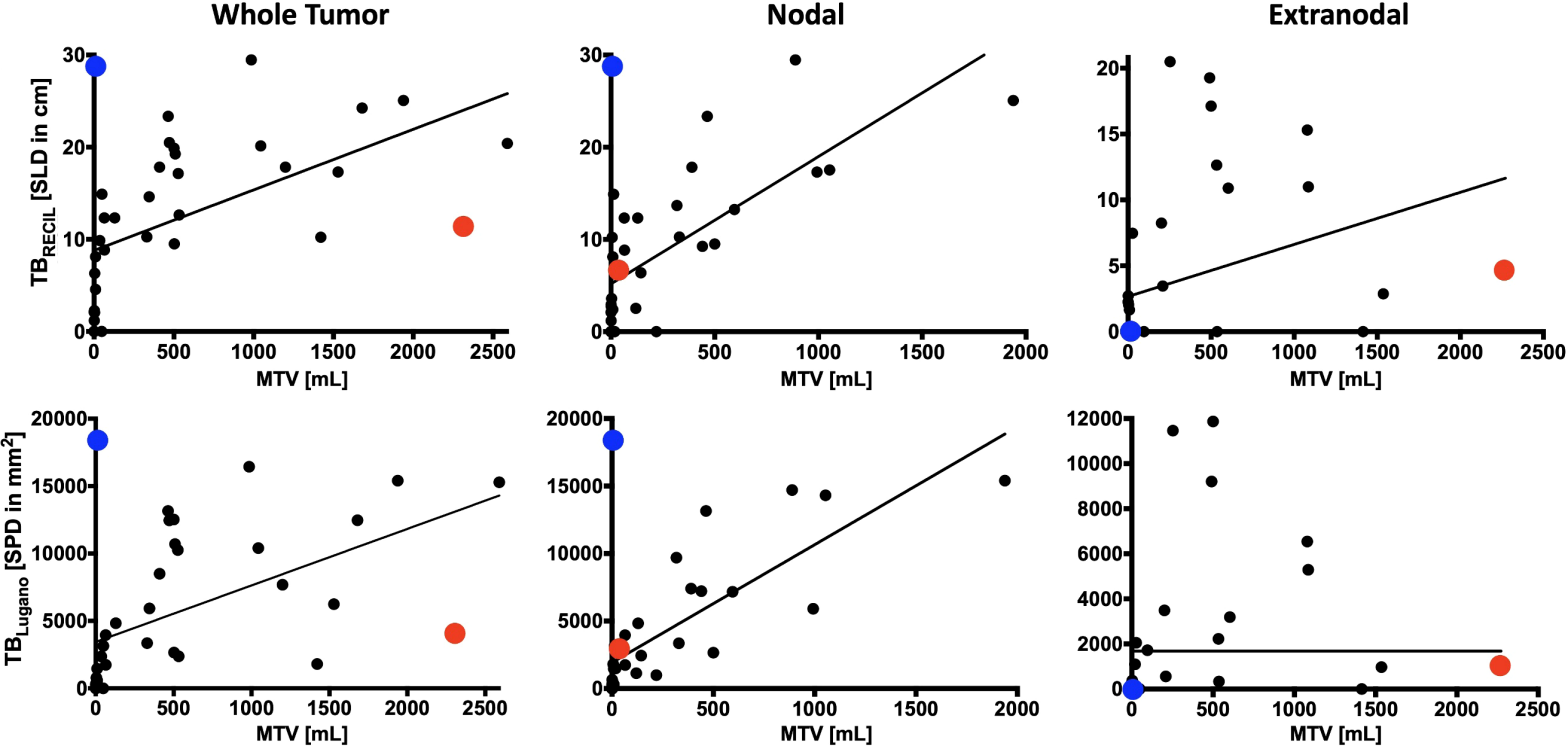
Comparison of RECIL, Lugano and Metabolic Tumor Burden Quantification. Depicted is the correlation of tumor burden at baseline PET/CT prior to CAR T-cell therapy according to RECIL (TB_RECIL_; **A**) and Lugano criteria (TB_Lugano_; **B**) on the y-axis to metabolic tumor volume (MTV) on the x-axis. Tumor burden is shown as whole tumor burden on the left, nodal tumor burden in the middle and extranodal tumor proportion on the right. An extreme outlier with high MTV to TB_RECIL_/TB_Lugano_ ratio was marked with red. Another outlier with high TB_RECIL_/TB_Lugano_ compared to MTV was marked with blue. The disease phenotypes of these two cases will be illustrated in **Figure 3**.

**Figure 3:**
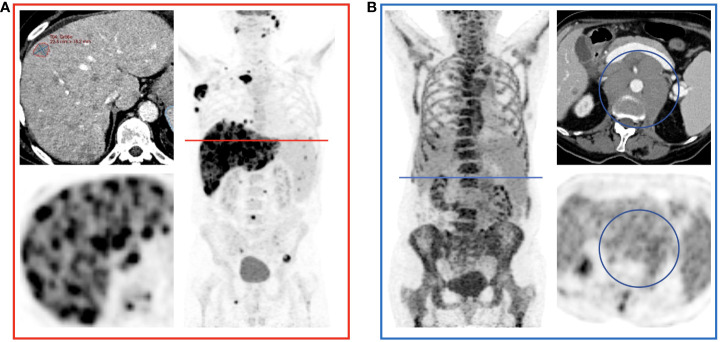
Examples of Over- and Underestimation with Metabolic Tumor Burden as Reference. PET/CT images of the patients color-coded in **Figure 2** are shown. **(A)** shows a patient with disseminated hepatic lymphoma manifestation in whom the tumor burden is underestimated by RECIL and Lugano compared to MTV due to the limited number of target lesions. **(B)** shows a patient after 4 cycles of bridging therapy in whom only small portions of the retroperitoneal lymphoma manifestation are hypermetabolic. Therefore, compared to MTV, a relatively high tumor burden is detected according to the RECIL and Lugano criteria.

The reference value for correlation analysis was set to TB_MTV_. The correlation of total TB_RECIL_ with total TB_MTV_ was ρ=0.744 (0.546-0.863, p<0.001), and total TB_Lugano_ with total TB_MTV_ ρ=0.741 (0.542-0.862, p<0.001). The correlation of TB_RECIL_ or TB_Lugano_ with TB_MTV_ was the lowest for extranodal disease. The results are illustrated in **Figure 2**. TB_RECIL_ and TB_Lugano_ displayed a strong positive correlation for total (ρ=0.983 [0.967-0.992], p<0.001), nodal (ρ=0.938 [0.880-0.968], p<0.001) and extranodal TB (ρ=0.896 [0.803-0.947], p<0.001). Patient examples for the largest deviations are depicted in **Figure 3**.

### Correlation analysis of TB metrics with LDH

The reference value for correlation analysis was set to LDH before apheresis (LDH_A_) and lymphodepletion (LDH_LD_) each. The correlation with LDH_A_ was low for total TB_RECIL_ (ρ=0.309, p=0.080), for total TB_Lugano_ (ρ=0.332, p=0.060), and especially for TB_MTV_ (ρ=0.156, p=0.380). LDH_LD_ showed a slightly better, yet moderate positive correlation for total TB_RECIL_ (ρ=0.515, p<0.001), for total TB_Lugano_ (ρ=0.521, p<0.001), and for TB_MTV_ (ρ=0.476, p=0.003).

### Survival analysis for different TB metrics

**Figure 4:**
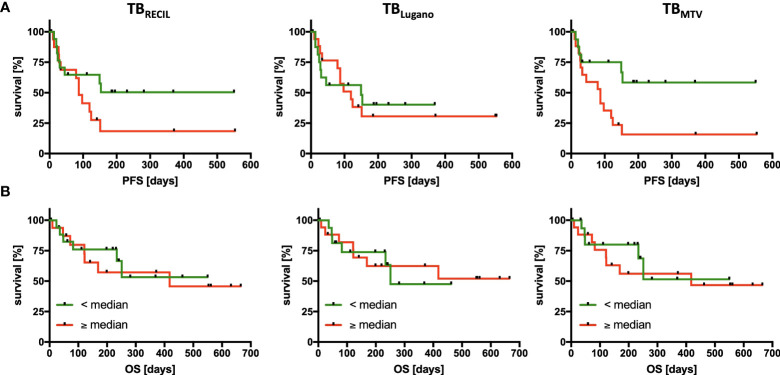
Association of Tumor Burden Metrics with Progression-Free and Overall Survival. PFS **(A)** and OS **(B)** analysis divided according to the 3 quantification methods of tumor burden by RECIL (left), Lugano (middle), and metabolic tumor volume (right). The median tumor burden was chosen as cut-off in each case with median baseline TB_RECIL_ 12.5 cm, TB_Lugano_ 4,030 mm^2^ and TB_MTV_ 330 mL. Patients with a tumor burden smaller than the median are labeled green and larger than the median are labeled red.

Median cutoff value for total TB_Lugano_ did not stratify PFS (HR=1.103, p=0.825) or OS (HR=0.923, p=0.888). Total TB_RECIL_ exhibited a non-significant trend for PFS (HR=1.963, p=0.124), but did not stratify OS (HR=1.206, p=0.733). The median cutoff value of 330 mL for TB_MTV_ significantly influenced PFS (HR=2.971, p=0.018), yet had no significant impact on OS in our study cohort (HR=1.286, p=0.652). The results are illustrated in **Figure 4**.

## Discussion

Lymphoma TB assessment before CART is gaining importance as a prognostic imaging biomarker. In this study, we investigated methods of TB assessment in the context of CD19 CART. Aside from the differences in absolute parameter values, which result from different measurement units, we have identified varying correlations between TB_RECIL_, TB_Lugano_ and TB_MTV_. These correlations were overall strong for TB_RECIL_ or TB_Lugano_ compared with TB_MTV_, and very strong for TB_RECIL_ compared with TB_Lugano_. Notably, both morphologic assessments of TB_RECIL_ or TB_Lugano_ demonstrated lower correlation than TB_MTV_ for extranodal disease, which itself represents a key prognostic marker (21).

Despite the numerically strong correlations among morphologic and metabolic methods, we identified several statistical outliers. We identified two patterns that led to the divergence in these patients: (1) disseminated lesions in extranodal disease, which led to underestimation by TB_RECIL_ or TB_Lugano_, and (2) metabolically inactive disease with large residual masses (e.g. after bridging therapy) that led to overestimation by TB_RECIL_ or TB_Lugano_ when compared with TB_MTV_. These potential reasons of divergence among TB metrics should be considered as potential sources of bias when comparing results from different trials.

Several trials have reported the impact of TB on PFS after CART. In the first CART trials, only few imaging-based parameters were analyzed. Regarding TB, the JULIET trial subjects had lower overall response rates (ORR) if the baseline TB_MTV_ exceeded 100 mL (2). In the ZUMA-1 trial (4), baseline TB according to Cheson (which can be considered identical to Lugano criteria) had the most pronounced prognostic impact on CART efficacy as assessed with durability of responses (6), yet no cutoff values were analyzed. The ZUMA-2 trial reported a 24% increase in ongoing responses for patients with TB_Lugano_ above the median (14). In CD30 CART of Hodgkin’s lymphoma, the PFS was 13.3 months longer if TB_MTV_ was below 60 mL (17).

Beyond trials, there have been reports on TB and impact on PFS and/or OS from retrospective studies that used commercial CART products. In two patient cohorts with DLBCL, Dean et al. showed that subjects with lower than median TB_MTV_ (147.5 mL) had superior PFS and OS with HRs of 0.40 and 0.25 respectively. In a smaller cohort, Iacoboni et al. equally identified that a high TB_MTV_ (≥ 25 mL) was associated with shorter PFS with a HR of 3.44 and indicated a trend towards shorter OS (8). The missing association between TB metrics and OS in our cohort may also result from the smaller sample size or differences in the study population. Other possible reasons may include, toxicity or treatment- and lymphoma-unrelated causes such as comorbidities, which should be investigated in larger samples.

The literature comparing different TB metrics in lymphoma is limited, yet particularly scarce in the setting before CART. This clinical setting impacts the morphologic and metabolic phenotypes of lymphoma in usually advanced disease stages. Prior treatments of pre-existing lymphoma manifestations may leave residual masses. These masses could be mistaken as part of the active lymphoma in morphologic imaging, whereas this does not affect the TB_MTV_. Yet, this can be easily avoided if prior imaging exams are available for comparison. In contrast with untreated lymphomas, the metabolic tumor extent in the clinical setting before CART infusion may also be altered by prior treatment lines or frequently used bridging regimens. Imaging-based response to bridging in particular can provide further valuable prognostic information (21).

The study by Dean et al. compared the baseline TB metrics TB_Lugano_ and TB_MTV_ in DLBCL patients before CART infusion (5). TB_MTV_ was determined using two different approaches of manual vs. semiautomated segmentation, which showed a moderate correlation. Here, the correlation between morphologic and metabolic TB assessment was low. As in the first study, we specifically analyzed the correlations regarding the whole tumor, nodal disease or extranodal disease. This indicated lower correlation between TB_Lugano_ and TB_MTV_ for extranodal disease. The low correlation in the study by Dean et al. may result from the high frequency of multiple extranodal sites (5).

Interestingly, the study by Dean et al. in the CART scenario had reported very similar SPD values for their two cohorts (44.3 cm^2^ vs. 43.0 cm^2^), yet the manual and semiautomated TB_MTV_ differed significantly (e.g. manual 147.5 mL vs. 72.8 mL; 6). This may have implications for randomized controlled trials. For example, the ZUMA-7 trial (15) reported only median SPD (i.e. TB_Lugano_) as an indicator of balance for the two study arms. One solution for future randomized controlled trials in CART may be the new International Metabolic Prognostic Index (IMPI) that incorporates age, stage and TB_MTV_. IMPI was recently shown to outperform the routinely applied International Prognostic Index (IPI) for PFS and OS in newly diagnosed DLBCL (22).

Our study has several limitations that should be considered when interpreting the results. First, we investigated a retrospective patient cohort with a limited sample size from a single institution, therefore the results may not be generalizable. Second, the precondition of a pre-CART PET-CT may lead to a bias for less severe disease stages as seriously ill patients with high treatment pressure may have undergone CT imaging more frequently. Third, we did not compare different software solutions to calculate TB_MTV_; yet the LIFEx method has been demonstrated to yield reproducible results compared with other approaches (19, 20).

In conclusion, TB metrics before CART vary significantly based on the method of assessment, which may impact their association with survival outcomes. The correlation between TB_RECIL_, TB_Lugano_ and TB_MTV_ was influenced by disease phenotype and prior bridging therapy. The TB methods of assessment must be considered when interpreting the impact of TB on outcomes in clinical trials. Considering the heterogeneity that can be observed in the current literature, our results argue for standardization and harmonization across centers.

## Data availability statement

The original contributions presented in the study are included in the article/**Supplementary Material**. Further inquiries can be directed to the corresponding author.

## Ethics statement

All medical records and imaging studies were reviewed with the approval of the LMU Munich Institutional Review Board (Ethikkommission der Medizinischen Fakultät der Ludwig-Maximilians-Universität München, Project Number 19-817) and informed patient consent. Histologic diagnoses were reviewed by expert pathologists. The patients/participants provided their written informed consent to participate in this study. Written informed consent was obtained from the individual(s) for the publication of any potentially identifiable images or data included in this article.

## Author contributions

MW and WK conceived and design the study. VLB, VB, KR, MR, MU, and CS collected the data. MW, VLB, VB, KR, and WK analyzed and interpreted the data. and MW and WK drafted the manuscript. KR, FD, PB, JR, MB-B, and MS revised the manuscript. All authors contributed to the article and approved the submitted version.

## Funding

The work was supported by funding from the research program “Förderung für Forschung und Lehre (FöFoLe) project number 1147” of the Medical Faculty of Ludwig Maximilian University (LMU) Munich and the Bavarian Cancer Research Center (BZKF).

## Conflict of interest

Author VB has received industry research support from Gilead, Novartis, Celgene, and Roche. Author KR declares having received research funding and travel support from Kite/Gilead and honoraria from Novartis. Author CS received travel support from Kite/Gilead. Author MB-B received research funding and honoraria from Novartis, Kite Pharma, Miltenyi Biotec, Mologen, MSD, Astellas, and Roche. Author MS received industry research support from Amgen, Gilead, Miltenyi Biotec, MorphoSys, Roche, and Seattle Genetics; served as a consultant or advisor to Amgen, Bristol Myers Squibb, Celgene, Gilead, Pfizer, Novartis, and Roche; is on the advisory boards of Amgen, Celgene, Gilead, Janssen, Novartis, Pfizer, and Seattle Genetics; and serves on the speaker’s bureau at Amgen, Celgene, Gilead, Janssen, and Pfizer.

The remaining authors declare that the research was conducted in the absence of any commercial or financial relationships that could be construed as a potential conflict of interest.

## Publisher’s note

All claims expressed in this article are solely those of the authors and do not necessarily represent those of their affiliated organizations, or those of the publisher, the editors and the reviewers. Any product that may be evaluated in this article, or claim that may be made by its manufacturer, is not guaranteed or endorsed by the publisher.
