# Supplementary material for: Lymphoma tumor burden before chimeric antigen receptor T-Cell treatment: RECIL vs. Lugano vs. metabolic tumor assessment
Source: Front Oncol. 2022 Sep 8;12:974029. doi: 10.3389/fonc.2022.974029 (PMC9492918; doi:10.3389/fonc.2022.974029)
Supplement: Supplementary file 1 [file DataSheet_1.pdf]

SUPPLEMENTARY MATERIAL

Supplementary Table 1: Correlation Analysis for RECIL, Lugano and Metabolic Tumor Burden.

TB Metric	Tumor proportion	Spearman ρ	95% CI	p-value
MTV vs. RECIL	Total<br>Nodal<br>Extranodal	0.744<br>0.763<br>0.660	0.546 - 0.863<br>0.577 – 0.874<br>0.419 – 0.814	<0.001
MTV vs. Lugano	Total<br>Nodal<br>Extranodal	0.714<br>0.852<br>0.749	0.542 – 0.862<br>0.725 – 0.923<br>0.554 – 0.866	<0.001
Lugano vs. RECIL	Total<br>Nodal<br>Extranodal	0.983<br>0.938<br>0.896	0.967 – 0.992<br>0.880 – 0.968<br>0.803 – 0.947	<0.001

Supplementary Figure 1: Different Dichotomization of Tumor Burden Metrics and Association with Progression-Free and Overall Survival.

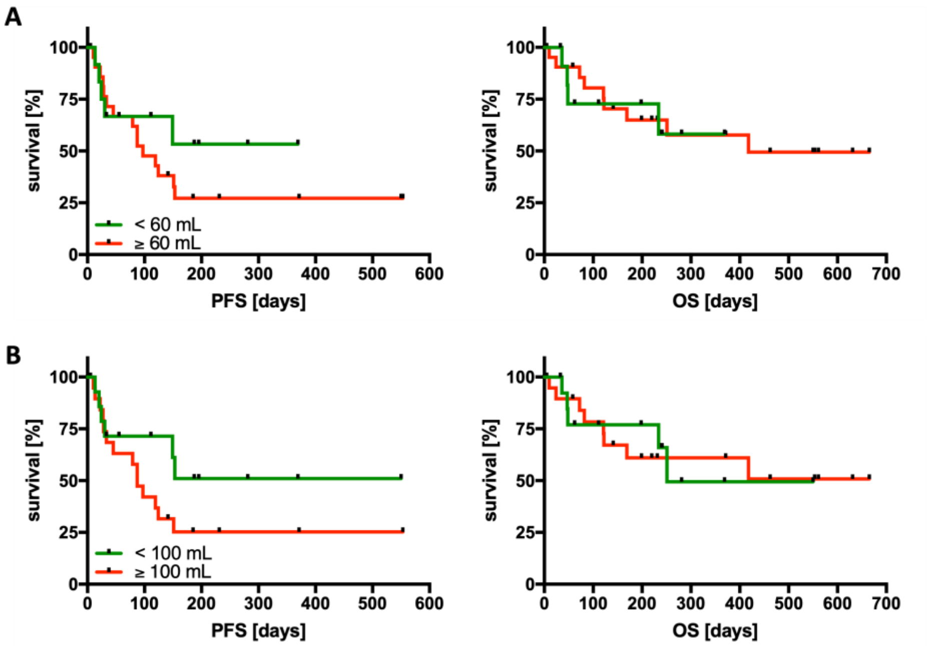

Kaplan-Meier survival curves for TB_{MTV} using the two dichotomization cut-offs 60 mL (**A**) and 100 mL (**B**) comparing PFS (on the left) and OS (on the right). Curves for patients with a tumor burden smaller than the
